# Short-term Effects of Nortriptyline on Sleep Parameters for Residual OSA after UPPP

**DOI:** 10.22038/ijorl.2025.87813.3947

**Published:** 2025

**Authors:** Reza Hosseini, Pedram Borghei, Amin Amali, Reihaneh Heidari, Reza Erfanian

**Affiliations:** 1 *Otorhinolaryngology Research Center, Tehran University of Medical Sciences, Tehran, Iran.*; 2 *Otorhinolaryngology Research Center, Tehran University of Medical Sciences, Amir Alam Hospital, Tehran, Iran, *; 3 *Otorhinolaryngology Research Center, Tehran University of Medical Sciences, Imam Khomeini Hospital, Tehran, Iran.*

**Keywords:** Obstructive sleep apnea, Nortriptyline, Uvulopalatopharyngoplasty, Sleep surgery, Residual OSA, Sleep pharmacotherapy, Daytime sleepiness

## Abstract

**Introduction:**

Uvulopalatopharyngoplasty (UPPP) surgery, though helpful, may not always achieve optimal results for obstructive sleep apnea (OSA). This prospective, uncontrolled trial investigates the potential of nortriptyline in improving symptoms and sleep parameters in OSA patients who have previously undergone UPPP.

**Materials and Methods:**

This single-center study evaluated the effects of nortriptyline in 24 OSA patients who had undergone UPPP surgery one year prior. Participants underwent a type IV sleep study one night before starting nortriptyline, and one month after treatment. The study employed the Stanford Subjective Snoring Scale (SSSS) and the Epworth Sleepiness Scale (ESS) to evaluate subjective reports of snoring and daytime sleepiness.

**Results:**

The Apnea-Hypopnea Index (AHI) significantly decreased from 16.8 to 11.4 events/hour (p-value = 0.02, effect size = 0.5). Mean oxygen saturation significantly increased from 94.1% to 95.4% (p-value = 0.016, effect size = 0.6). Time spent below 90% oxygen saturation significantly decreased from 7.2% to 4.8% (p-value = 0.007, effect size = 0.73). ESS significantly decreased from 9.6 to 7.2 (p-value < 0.001, effect size = 0.89). SSSS significantly decreased from 7.1 to 3.1 (p-value = 0.002, effect size = 0.90). Minor side effects were monitored; one participant developed excessive sleepiness, and another participant reported heart palpitations.

**Conclusion:**

This trial suggests that nortriptyline may be a promising treatment option for improving residual sleep apnea in patients who have already undergone UPPP but still experience symptoms. Further research is needed to confirm these initial results. Trial registration number: IRCT20230614058482N1

## Introduction

Obstructive sleep apnea (OSA) is a chronic sleep disorder characterized by recurrent pauses in breathing during sleep due to upper airway obstruction (1). Untreated OSA can lead to a wide range of clinical consequences. These include daytime hypersomnolence, neurocognitive dysfunction, cardiovascular disease, metabolic dysfunction, pulmonary hypertension, and poor symptom control in asthma and chronic obstructive pulmonary disease (COPD) (2). Continuous Positive Airway Pressure (CPAP) is an effective treatment for OSA. CPAP therapy has been shown to significantly reduce the apnea-hypopnea index (AHI) and oxygen desaturation index (ODI) in patients with OSA (3). 

Compliance with CPAP treatment for obstructive sleep apnea (OSA) is a significant issue, with rates ranging from 30-80% (4). Despite overcoming compliance challenges associated with CPAP, Uvulopalato- pharyngoplasty (UPPP) for obstructive sleep apnea (OSA) may not always achieve optimal results (5). Several approaches have been investigated to improve the outcomes of UPPP, including its combination with hyoid suspension surgery (6), radiofrequency tongue base reduction (7), and the application of oral appliances (8). Pharmacotherapy plays a potential role in the treatment of OSA. Recent insights into the pathophysiology of OSA have led to the identification of distinct subgroups and potential drug targets (9). These targets include impaired upper airway anatomy, ineffective upper airway dilator muscle function, low respiratory arousal threshold, and unstable breathing control (10). Several drug candidates have shown promising results in reducing sleep-disordered breathing in clinical trials (11). 

Nortriptyline, a tricyclic antidepressant, has been studied for its effects on various conditions. Its potential impact on OSA may stem from its modulation of serotonin and norepinephrine, which influence sleep regulation and airway muscle tone. Studies show this agent acutely and persistently decreased REM sleep, increased phasic REM activity, and decreased sleep apnea, without affecting periodic limb movements during sleep. (12). It has been shown to have anti-inflammatory properties (13). It has been found to have a generally safe profile in several studies (14). However, despite the known challenges with post-UPPP OSA and the potential pharmacological benefits of nortriptyline on sleep regulation and airway muscle tone, there is a notable lack of research specifically evaluating the efficacy of nortriptyline in patients with residual OSA after UPPP surgery. This study aims to address this gap by evaluating the effectiveness of nortriptyline in reducing residual symptoms and improving objective parameters in patients with obstructive sleep apnea who have previously undergone UPPP surgery.

## Materials and Methods

**Fig 1:**
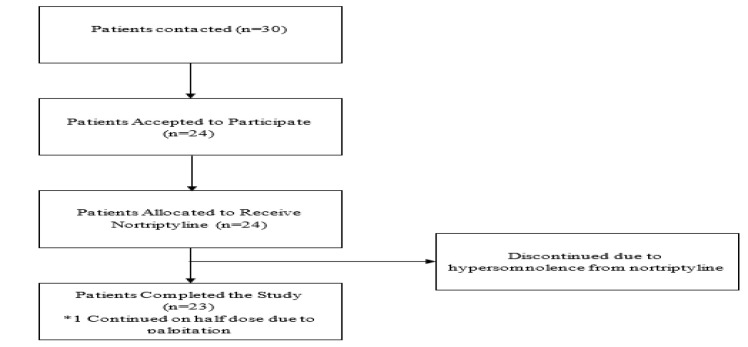
Consort Flow Diagram for Patient Enrollment and Retention.

Study Design and Participants: This single-center, open-label, prospective trial enrolled 24 participants between October 2023 and February 2024 to evaluate the effects of nortriptyline on sleep parameters and quality-of-life measures. Participants were diagnosed with obstructive sleep apnea (OSA) and had undergone uvulopalatopharyngoplasty (UPPP) with hyoid suspension one year before study inclusion (Figure 1). Details of the surgical procedure are described elsewhere (6).

The study was registered at the clinical registry system (IRCT20230614058482N1) and received ethical approval from the local ethics committee (IR. TUMS. AMIRALAM. REC.1402.006). Informed consent was obtained from all participants, and the study adhered to the Declaration of Helsinki principles.

### Pre-operative Polysomnography:

All participants underwent a level 1 sleep study (polysomnography) before the initial surgery. This study recorded sleep parameters, including the apnea-hypopnea index (AHI), mean oxygen saturation, time spent below 90% oxygen saturation (T90%), and lowest oxygen desaturation.

Demographic and Subjective Measures: Age, sex, and body mass index (BMI) were documented at admission. Additionally, participants completed the Stanford Snoring Subjective Snoring Scale (SSSS) (15) and the Epworth Sleepiness Scale (ESS) (16) to assess their subjective experience of snoring and daytime sleepiness, respectively.

### Pre-Nortriptyline Sleep Study:

One night before initiating nortriptyline treatment, a level 4 sleep study was conducted using the Berry-BM2000A wearable device. This study measured the AHI, mean oxygen saturation, T90%, and lowest oxygen desaturation.

### Nortriptyline protocol:

Participants received oral nortriptyline starting with a dose of 10 milligrams, used two hours before bedtime, for one week. The dosage was then increased to 20 milligrams, also taken two hours before bedtime. If participants experienced minor side effects and could not tolerate the 20-milligram dose, the dosage was reduced back down to 10 milligrams. The treatment duration was one month.

### Follow-up measures:

One month after initiating nortriptyline treatment, BMI, SSSS, and ESS were reassessed. A follow-up level 4 sleep study was conducted using the same wearable device. This study measured AHI, mean oxygen saturation, T90%, and lowest oxygen desaturation. Participants continued taking nortriptyline during this study. Sleep parameters measured by the wearable device were used to assess the objective effects of nortriptyline.

### Statistical analysis:

Statistical analyses were performed using JASP (Version 0.18.1). The normality of data distribution for measured parameters (Apnea-Hypopnea Index, mean oxygen saturation, time spent below 90% oxygen saturation, Epworth Sleepiness Scale, Stanford Subjective Snoring Scale, and body mass index) was assessed using the Shapiro-Wilk test. For normally distributed variables, paired Student’s t-tests were used to compare pre- and post-nortriptyline treatment outcomes. For non-normally distributed variables, Wilcoxon signed-rank tests were applied. Effect sizes were calculated using Cohen’s d for t-tests and matched rank-biserial correlation for Wilcoxon tests. A p-value < 0.05 was considered statistically significant.

## Results

***Results***

One participant developed excessive sleepiness and discontinued nortriptyline use, subsequently exiting the study. Another participant reported heart palpitations. After consulting a cardiologist, they reduced their nortriptyline dosage by half and continued the study due to perceived benefits.

A total of 23 patients were included in the study, with 5 females and 18 males. The mean age of the participants was 39.8 years old, with a standard deviation of 8.1 years. The median age was 39 years old, and the interquartile range (IQR) was 10 years. 

### Preoperative features:

The mean BMI was 28.5 with a standard deviation of 2.5. The median BMI was 29.1, and the interquartile range (IQR) was 2.7. The mean ESS score was 11.1 with a standard deviation of 5.6. The median ESS score was 9, and the IQR was 10. The mean SSSS was 7.1 with a standard deviation of 2.8. The median SSSS was 8, and the IQR was 4.

The mean AHI was 48.4 events per hour, with a standard deviation of 24.1 events/hour. The median AHI was 47 events/hour, with an interquartile range (IQR) of 47.6 events/hour. Other preoperative polysomnographic characteristics are detailed in Table 1.

**Table 1:** Preoperative polysomnographic features of participants. AHI: Apnea-Hypopnea Index, SD: Standard deviation, IQR: Interquartile range, T90%: Time spent below 90% oxygen saturation.

	**Pre-operative AHI**	**Pre-operative Mean O2%**	**Pre-operative Lowest O2%**	**Pre-operative T90%**
Median	47	92	77	7.6
Mean	48.4	91.0	73.3	19.1
SD	24.14	4.0	12.2	22.9
IQR	45.6	3.5	17.5	22.6

### Baseline Features of Participants Before Nortriptyline Initiation (1 Year After Surgery)

The mean BMI was 29.0 with a standard deviation of 2.5. The median BMI was 29.5, and the interquartile range (IQR) was 2.4. 

The mean ESS score was 9.6 with a standard deviation of 6.0. The median ESS score was 8, and the IQR was 9. 

The mean SSSS was 5.0 with a standard deviation of 3.6. The median SSSS was 5, and the IQR was 7.

The mean AHI was 16.8 events per hour, with a standard deviation of 12 events/hour. The median AHI was 12.5 events/hour, with an interquartile range (IQR) of 14.4 events/hour. Other preoperative polysomnographic characteristics are detailed in (Table 2).

**Table 2:** Wearable sleep study parameters before and after using nortriptyline. AHI: Apnea-Hypopnea Index, SD: Standard deviation, IQR: Interquartile range, T90%: Time spent below 90% oxygen saturation

	**Pre-Nortriptyline AHI**	**Post-Nortriptyline AHI**	**Pre-Nortriptyline Mean O2%**	**Post-Nortriptyline Mean O2%**	**Pre-Nortriptyline Lowest O2% saturation**	**Post-Nortriptyline Lowest O2% saturation**	**Pre-Nortriptyline T90%**	**Post-Nortriptyline T90%**
Median	12.48	10.25	95	95	86	88	0.17	0.12
Mean	16.8	11.4	94.1	95.4	85.6	87.4	7.2	4.8
SD	12.0	1.0	2.5	2.6	5.4	5.6	16.6	15.4
IQR	14.38	9.00	1.5	2	7	5.5	4.7	1.6
Effect size	0.51		0.64		0.4		0.73	
P value	0.02		0.016		0.07		0.007	

### Impact of nortriptyline on BMI:

Participants receiving nortriptyline treatment experienced a statistically significant increase in BMI two months after initiation (p=0.02). The mean BMI rose to 29.4 (SD = 2.8), with a median of 29.6 and an interquartile range (IQR) of 2.6. The effect size was 0.64.

### Impact of nortriptyline on ESS:

Participants receiving nortriptyline treatment experienced a statistically significant decrease in ESS two months after initiation (p<0.001). The mean ESS decreased to 7.2 (SD = 5.8), with a median of 5 and an interquartile range (IQR) of 8. The effect size was 0.89.

### Impact of nortriptyline on SSSS:

Participants receiving nortriptyline treatment experienced a statistically significant decrease in SSSS two months after initiation (p=0.002). The mean SSSS decreased to 3.1 (SD = 2.8), with a median of 2 and an interquartile range (IQR) of 4.5. The effect size was 0.90. 

### Impact of nortriptyline on sleep apnea severity:

- Apnea-Hypopnea Index (AHI): AHI significantly decreased to 11.4 events per hour (SD = 1.0) from baseline, with a p-value of 0.02 and an effect size of 0.51.
- Mean Oxygen Saturation: Mean oxygen saturation significantly increased to 95.4% (SD = 2.7) from baseline, with a p-value of 0.016 and an effect size of 0.61.
- Lowest Oxygen Saturation: While not statistically significant (p-value = 0.070), the lowest recorded oxygen saturation showed a slight non-significant increase.
- Time Spent Below 90% Oxygen Saturation (T90%): T90% significantly decreased to 4.8% (SD = 15.4) from baseline, with a p-value of 0.007 and an effect size of 0.73.

Further details of sleep study parameters, including baseline values, are available in Table 2.

## Discussion

This is the first study to investigate the potential pharmacologic benefits of nortriptyline specifically in patients who have undergone prior sleep surgery. The study provides compelling evidence that nortriptyline can significantly improve both subjective and objective outcomes in sleep surgery patients. The observed improvements across various measures, including snoring loudness, sleepiness, oxygenation, and apnea severity, highlight the potential of nortriptyline as a therapeutic option in this population. 

Nortriptyline, a tricyclic antidepressant, has been found to have various effects on sleep. It has been shown to suppress REM sleep and increase delta-wave production and density in the first non-REM period in depressed people (17). In elderly patients with bereavement-related depression, nortriptyline was associated with improved sleep quality, REM percent, REM latency, REM density, and delta sleep ratio (18). Furthermore, nortriptyline has been found to decrease REM sleep, increase phasic REM activity, and decrease sleep apnea, with these effects being associated with a reduced likelihood of depression recurrence (12). 

While these findings suggest a potential mechanism for nortriptyline’s effect on sleep apnea, it is important to acknowledge that our study utilized Level 4 sleep monitoring, which lacks the ability to evaluate sleep stages, arousals, or sleep architecture. Therefore, the precise mechanism by which nortriptyline affects sleep-disordered breathing cannot be objectively confirmed. Our statements regarding REM suppression and delta sleep increases are, therefore, speculative and based on prior literature rather than direct observation in this cohort.

REM sleep is characterized by decreased muscle tone, leading to a heightened risk of upper airway collapse and apneas (19). By reducing REM sleep duration, nortriptyline might decrease obstructive sleep apnea severity in the current study. Delta sleep (also called N3 and slow wave sleep) promotes increased arousal thresholds (20) and more stable breathing patterns due to stronger muscle activity (21). Nortriptyline-induced N3 sleep increase may enhance upper airway muscle tone and stability, elevate arousal thresholds, and consequently reduce the severity of obstructive sleep apnea. While the exact mechanisms remain elusive, this study's observed improvements in sleep apnea severity following nortriptyline treatment might be partially explained by its interaction with both serotonin and norepinephrine reuptake. Serotonin (5-hydroxytryptamine or 5-HT) exhibits complex effects on respiratory control, stimulating central serotonin receptors would stimulate respiration (22,23). However, stimulating peripherally located serotonin receptors often has an inhibitory effect (24). It has been suggested that noradrenergic loss plays a more prominent role in sleep-related muscle reduction than serotonin (around 10%), both systems offer potential targets for improving upper airway patency (25). Nortriptyline's ability to inhibit the reuptake of both noradrenaline and serotonin could modulate central nervous system breathing pathways, potentially enhancing upper airway muscle response and contributing to the observed reduction in apnea events. Further research is needed to elucidate the specific receptor subtypes and neural circuits involved in nortriptyline's effect on sleep apnea severity.

While anticholinergic medications like oxybutynin have been explored for their potential to improve obstructive sleep apnea (OSA), their effectiveness and mechanisms remain under investigation. Oxybutynin exerts its effects by blocking muscarinic receptors in the hypoglossal motor nucleus and enhancing breathing muscles (26). Nortriptyline also exhibits central anticholinergic properties (27) and may explain some beneficial effects of this medicine in our study.

A combination of atomoxetine (a selective norepinephrine reuptake inhibitor) and oxybutynin (Antimuscarinic agent) has shown promising results in reducing the severity of obstructive sleep apnea (OSA) in various studies (28). Previous research suggests the atomoxetine-oxybutynin combination might be beneficial for obstructive sleep apnea (OSA) treatment. This study highlights its potential by demonstrating that it significantly improved upper airway collapsibility**. **However, the study also suggests that patients with lower AHI and less severe collapsibility benefit most from this therapy (29). Given its dual noradrenergic and anti-muscarinic effects, nortriptyline might be helpful for a specific OSA subgroup. Our patients were previously treated with sleep surgery with improved airway anatomy and reduced OSA severity. The drug's dual action might offer additional benefits for these specific patients with an acceptable safety profile (14).

Nortriptyline, as a tricyclic antidepressant, has been found to have anti-inflammatory effects. This is attributed to the tricyclic antidepressant's ability to inhibit catecholamine uptake, leading to a potentiation of catecholamine response (30). Additionally, tricyclic antidepressants have been shown to decrease the production of nitric oxide, a key player in inflammation (31). These findings suggest that nortriptyline may have the potential as an anti-inflammatory agent. Nortriptyline's anti-inflammatory effects on peripheral tissues like the upper airway muscles may reduce inflammation-induced swelling and improve airway patency and muscle function. While these anti-inflammatory properties are interesting, it is crucial to note that our study did not directly measure inflammatory markers, and therefore, these effects are not directly supported by our data.

Treatment with the tricyclic antidepressant nortriptyline was associated with moderate weight gain. Several mechanisms might contribute to nortriptyline-induced weight gain. Nortriptyline can stimulate appetite, leading to increased calorie intake. The drug might reduce energy levels, potentially leading to decreased physical activity and lower calorie expenditure (32). Excess weight is the strongest factor in the risk and severity of OSA, and weight loss is associated with decreased OSA severity (33). Our study identified a significant increase in BMI among participants treated with nortriptyline for OSA. While nortriptyline may improve OSA, the observed increase in BMI could potentially counteract these benefits. While nortriptyline may improve OSA, the observed increase in BMI could potentially counteract these benefits and warrants a more critical discussion regarding its long-term therapeutic goals in OSA management. This finding is particularly concerning given the strong association between weight gain and OSA severity.

While nortriptyline, a secondary amine tricyclic antidepressant, possesses some activating properties, its overall effect on sleepiness can be complex and multifaceted (34). Our study findings suggest that nortriptyline improved sleepiness as measured by the ESS in patients with OSA. This finding may be because of improved OSA and decreased sleep fragmentation. Rigorous controlled clinical trials with objective sleep measures like polysomnography are needed to definitively understand nortriptyline's impact on sleepiness in OSA. 

While our study provides insights into the potential benefits of nortriptyline for improving sleepiness in OSA patients after sleep surgery, there are limitations that warrant consideration. The absence of a control group makes it difficult to definitively attribute the observed improvements to nortriptyline, as other factors or natural fluctuations in sleepiness could also contribute. Additionally, the short follow-up period limits our ability to assess the long-term sustainability of these effects. Furthermore, the lack of polysomnography data prevents a deeper understanding of nortriptyline's impact on sleep architecture and objective sleep parameters, which could provide valuable insights into the mechanisms underlying its observed effects. Addressing these limitations in future studies with robust designs, longer follow-up periods, and objective sleep measures would strengthen the evidence base and provide more definitive conclusions about nortriptyline's potential role in OSA management.

## Conclusion

This study suggests nortriptyline may improve residual sleep apnea after surgery, showing short-term reductions in AHI, improved oxygen saturation, and decreased daytime sleepiness and snoring. While promise

ng, limitations include a small sample, limited diagnostics (Level 4 sleep monitoring), and no control group, requiring cautious interpretation. Future research needs randomized controlled trials with placebo arms, longer follow-up, and polysomnography.
